# Clinical features and molecular genetic investigation of infantile-onset ascending hereditary spastic paralysis (IAHSP) in two Chinese siblings caused by a novel splice site *ALS2* variation

**DOI:** 10.1186/s12920-024-01805-x

**Published:** 2024-01-31

**Authors:** Qiang Zhang, Qi Yang, Jingsi Luo, Xunzhao Zhou, Shang Yi, Shuyin Tan, Zailong Qin

**Affiliations:** 1 Maternal and Child Health Hospital of Guangxi, Nanning, China; 2 Guangxi Key Laboratory of Precision Medicine for Genetic Diseases, The Maternal and Child Health Care Hospital of Guangxi Zhuang Autonomous Region, Nanning, China; 3 Guangxi Key Laboratory of reproductive health and birth defect prevention, The Maternal and Child Health Care Hospital of Guangxi Zhuang Autonomous Region, Nanning, China; 4 Guangxi Clinical Research Center for Pediatric Diseases, The Maternal and Child Health Care Hospital of Guangxi Zhuang Autonomous Region, Nanning, China

**Keywords:** Infantile-onset ascending hereditary spastic paralysis, IAHSP, *ALS2*, Whole-exome sequencing, Minigene

## Abstract

**Objective:**

*ALS2*-related disorder involves retrograde degeneration of the upper motor neurons of the pyramidal tracts, among which autosomal recessive Infantile-onset ascending hereditary spastic paralysis (IAHSP) is a rare phenotype. In this study, we gathered clinical data from two Chinese siblings who were affected by IAHSP. Our aim was to assess the potential pathogenicity of the identified variants and analyze their clinical and genetic characteristics.

**Method:**

Here, Whole-exome sequencing (WES) was performed on proband to identify the candidate variants. Subsequently, Sanger sequencing was used to verify identified candidate variants and to assess co-segregation among available family members. Utilizing both silico prediction and 3D protein modeling, an analysis was conducted to evaluate the potential functional implications of the variants on the encoded protein, and minigene assays were performed to unravel the effect of the variants on the cleavage of pre-mRNA.

**Results:**

Both patients were characterized by slurred speech, astasia, inability to walk, scoliosis, lower limb hypertonia, ankle clonus, contracture of joint, foot pronation and no psychomotor retardation was found. Genetic analysis revealed a novel homozygous variant of *ALS2*, c.1815G > T(p.Lys605Asn) in two Chinese siblings. To our knowledge, it is the first confirmed case of a likely pathogenic variant leading to IAHSP in a Chinese patient.

**Conclusion:**

This study broadens the range of *ALS2* variants and has practical implications for prenatal and postnatal screening of IAHSR. Symptom-based diagnosis of IAHSP is frequently difficult for medical practitioners. WES can be a beneficial resource to identify a particular disorder when the diagnosis cannot be determined from the symptoms alone.

**Supplementary Information:**

The online version contains supplementary material available at 10.1186/s12920-024-01805-x.

## Introduction

Amyotrophic lateral sclerosis (ALS) in its entirety affects 4.42 per 100,000 people worldwide, ALS is largely sporadic, but in 5–10% of the cases, the disease is inherited through autosomal dominant or recessive genetic variants [1, 2]. *ALS2* was initially identified as a form of ALS in a large Tunisian family with a history of consanguinity [3]. Autosomal recessive variants in the *ALS2* gene have been linked to a variety of disorders. These diseases can manifest with a clinical continuum from Infantile Ascending Hereditary Spastic Paraplegia (IAHSP) to Juvenile Primary Lateral Sclerosis (JPLS), and Juvenile Amyotrophic Lateral Sclerosis (JALS) [4]. The first two disorders affect only the upper neurons, while the latter affects both the upper and lower neurons. There may be overlap in clinical presentation across these disease subgroups, with IAHSP and JPLS sometimes being used interchangeably. IAHSP was initially reported in 1996 in 3 Kuwaiti children born of a consanguineous parentage [5]. The prevalence of IAHSP disorders is unknown, with only a few cases having been described in a variety of ethnic backgrounds. To date, most of the reported cases of IAHSP have been from Mediterranean and Asia countries [5]. IAHSP is caused by a variant in the *ALS2* gene, encoding for the Alsin protein. Alsin protein is expressed in the central nervous system and non-nervous tissues, with the cerebellum and kidney showing the highest enrichment and the spinal cord and heart showing the lowest [6, 7]. *ALS2* gene is located on chromosome 2q33, and is composed of 33 introns and 34 exons. There are at least two transcripts long (6.5 kb) and short (2.6 kb) and 13 splice variants [8]. It also contains a few signalling domains and protein trafficking domains. The structure of alsin predicts that it functions as a guanine nucleotide exchange factor (GEF). GEFs regulate the activity of members of the Ras superfamily of GTPases. Alsin plays a role in endosomal and mitochondrial trafficking as well as cytoskeleton maintenance and endocytosis [9]. How alsin variants lead to the pathology is still unclear. Indeed, preliminary genotype-phenotype correlations suggested that the truncation of full-length alsin, and therefore its loss of function, account for the upper motor neurons (UMN) degeneration, whereas the short variant, and possibly loss of both full-length and short forms of ALS2, might be related to lower motor neurons (LMN) defects [8]. Here, our study reports a novel variant in the *ALS2* gene, which is the first confirmed case of a likely pathogenic variant leading to IAHSP in a Chinese patient.

## Materials and methods

### Next generation sequencing

Both patients were examined by WES. Genomic DNA samples were collected and sequence libraries were constructed using the Agilent Sure Select Human Whole Exome V2 Kit (Agilent Technologies, Santa Clara, CA). Prepared libraries were sequenced using the HiSeq2500 System (Illumina, San Diego, CA). Reads obtained from the BWA software package (v. 0.7.15) was mapped with the human reference genome (GRCh37/hg19). Variant calling and variant annotation were performed using the Genome Analysis Toolkit (GATK) and variant annotation and prioritization were performed using TGex software (LifeMap Sciences, Inc.v5.7).

### Sanger sequencing confirmation

A 2.5 ml of venous blood sample was taken from the other family members. Sanger sequencing was performed to confirm the variant in proband and its family members. The following primers designed by oligo7 were used: 5′-AACACGTGGCTTCCTGTTTT-3′, and 5′-TGCAAAATCAGATTCACAACG-3’for c.1815G > T(p.Lys605Asn) .

### Bioinformatic analysis and verification of observations

The bioinformatics tools SIFT (http://sift.jcvi.org/), Variant Taster software (http://www.varianttaster.org/), PolyPhen-2 (http://genetics.bwh.harvard.edu/pph2/), Combined Annotation Dependent Depletion (CADD) (https://cadd.gs.washington.edu), and varSEAK (https://varseak.bio/index.php) were applied to predict the impact of variants on protein function. The protein 3D structures of ALS2 were generated by Swiss-Model server (https://swissmodel.expasy.org/), and the ACMG/AMP variant classification guidelines were employed for variant classification [10].

### Minigene splicing assay

We utilized minigene technology to validate in vitro whether the c.1815G > T mutation affects pre-mRNA splicing. We synthesized both a wild-type DNA sequence and a mutant-type DNA sequence based on the candidate pathogenic variant. According to the references, our synthesis only included the 200 bp region flanking the exon for the intron region [11]. The DNA was then cleaved with the cloning vector pcDNA3.1 at the HindIII/BamHI digestion site. The fragment of interest was inserted into a human cloning vector by recombination reaction, and the recombinant product was transformed into competent cells and cultured. We selected correctly recombinant wild-type and variant plasmids, transfected them into 293 T cells, and obtained RNA cDNA by reverse transcription. The primers pcDNA3.1-F: 5′-CTAGAGAACCCACTGCTTAC-3′ and pcDNA3.1-R: 5′-TAGAAGGCAGTCGAGG-3′ were used to amplify the sequence, detect them by gel electrophoresis, and finally sequence the recovered gel product.

## Results

### Clinical data

**Fig. 1:**
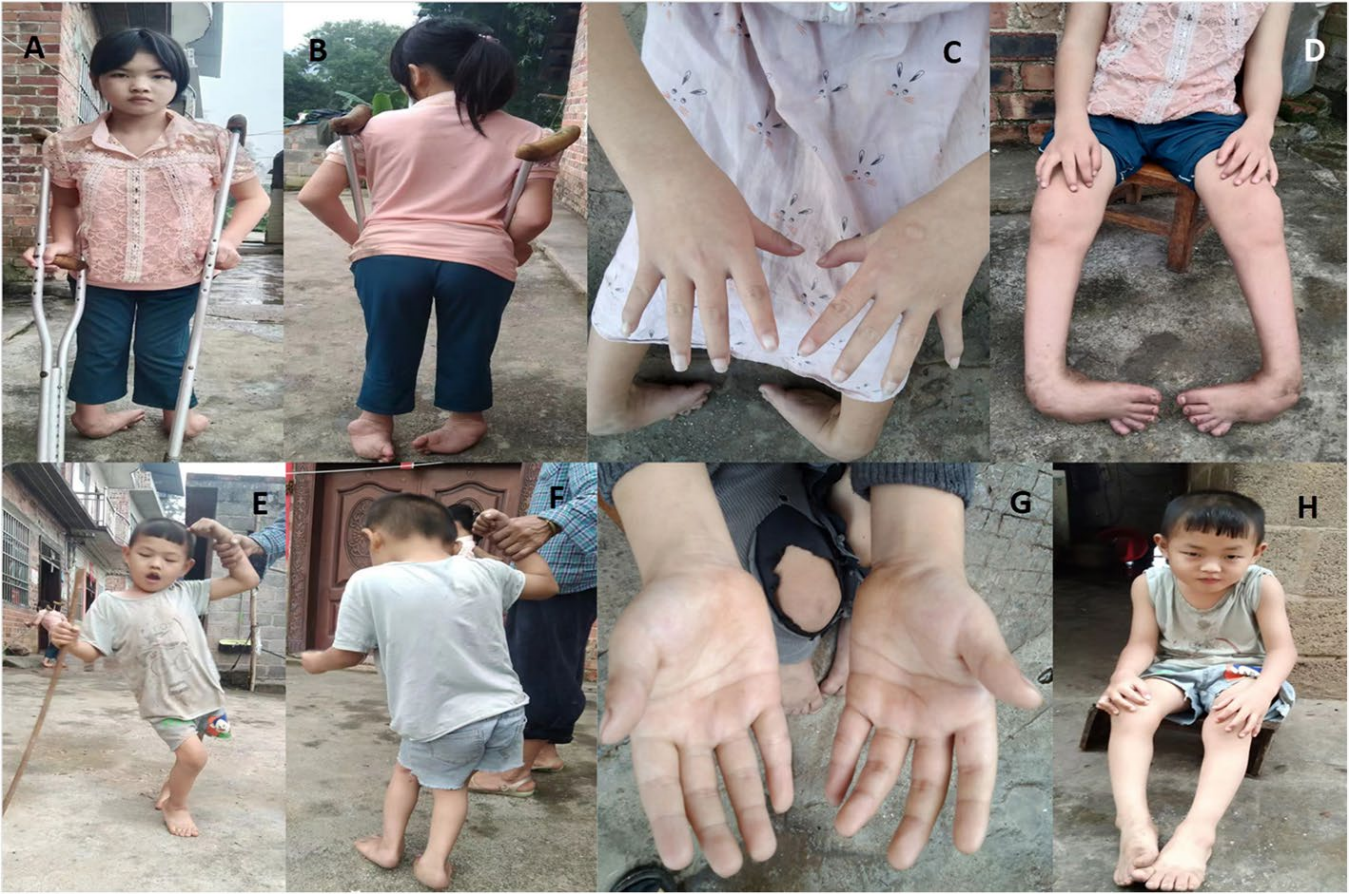
The clinical features of the patient with Infantile-onset ascending hereditary spastic paralysis. **A**-**D** show the phenotypic characteristics of sister; **E**-**H** show the phenotypic characteristics of brother: astasia, inability to walk, scoliosis, contracture of joint,lower limb hypertonia, foot pronation

The two Chinese siblings presented to our hospital with functional motor deficits at the ages of 5.6 years and 11 years. Both were born at full term to non-consanguineous parents, and there were no signs of neonatal asphyxia during delivery. They had normal Apgar scores of 10/10/10 and developed normally until the age of 2. During the physical examination, we collected data on the patients’ (sister-brother) height (130 cm [< 2 SD], 105 cm [< 2 SD]), weight (35 kg, 17.5 kg), and head circumference (51.1 cm, 50.3 cm). We observed slurred speech, astasia, inability to walk, scoliosis, lower limb hypertonia, ankle clonus (+), joint contractures, foot pronation, an adductor angle of 30°, a popliteal angle of 100°, and a dorsiflexion angle of 120° (Fig. 1). They had a scissor gait when walking with support. Ultrasonography of the hepatobiliary-pancreatic-splenic system was normal, as were magnetic resonance imaging (MRI) and neonatal echocardiography.

### Genetic testing

**Fig. 2:**
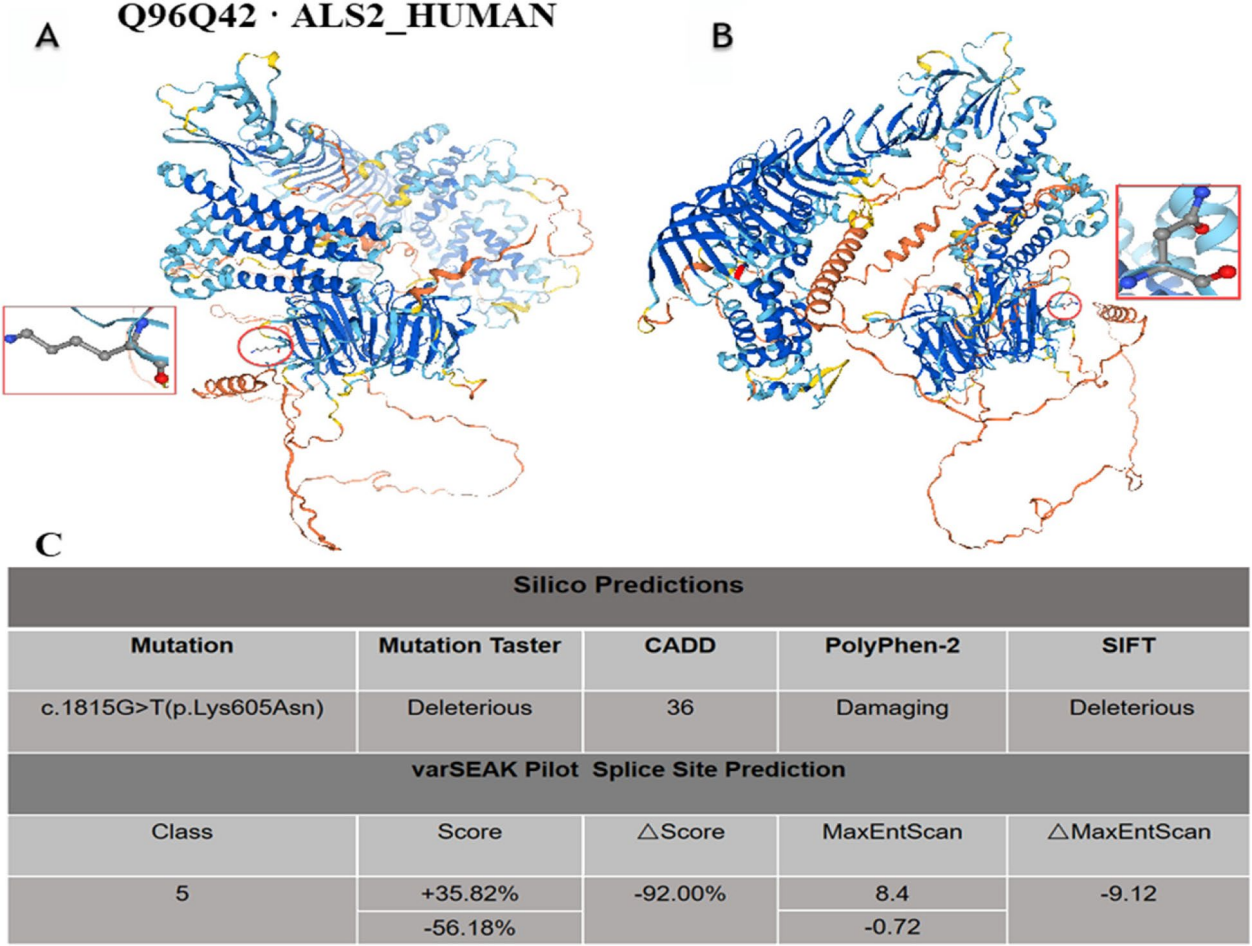
**A**-**B **Three-dimensional structures of ALS2 [(**A**): wild-type, (**B**):c.1815G > T(p.Lys605Asn)mutant-type]; **C **in silico predictions. The impact of both of the ALS2 variants was predicted using five in silico tools

**Fig. 3:**
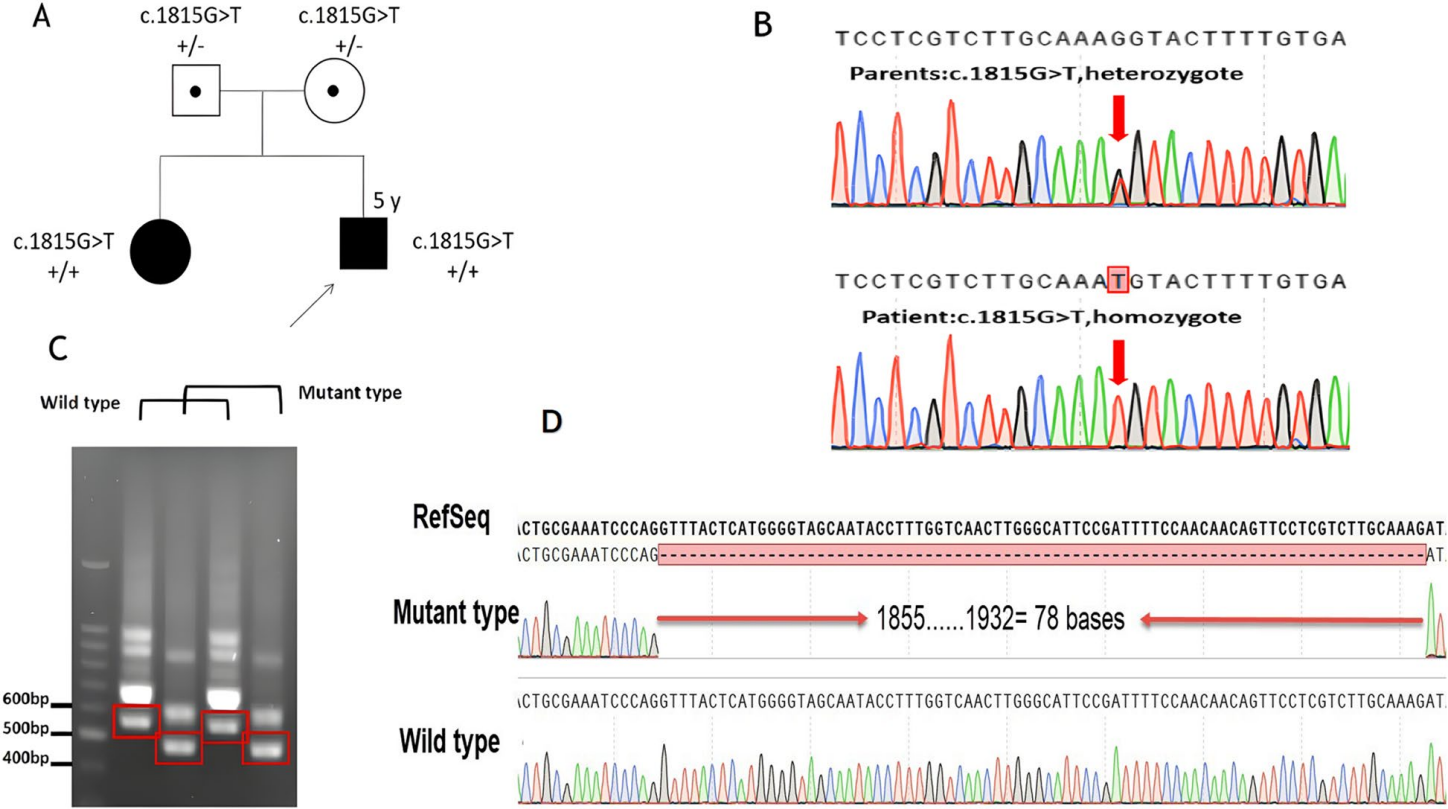
**A** family pedigree, Circles denote females; squares denote males; black square denotes affected male, and black circle denotes affected female; a dot in the middle of a shape indicates a heterozygous carrier; arrow indicates the proband. **B **Sanger sequencing result. **C** and **D **The results of agarose gel electrophoresis and DNA sequence analysis

We identified a homozygous variant, c.1815G > T (p.Lys605Asn), in the *ALS2* gene (NM_020919.3, Chr2:202614435 in exon 8) in the proband (a boy) using WES. Sanger sequencing confirmed that his sister also carries c.1815G > T (p.Lys605Asn), and the variant was inherited from both parents (see Fig. 3, A and B). We used four in silico tools to predict the impact of the novel variants (see Fig. 2C), which suggested that c.1815G > T (p.Lys605Asn) was a harmful variant. The SWISS-MODEL software was employed to perform a comparative analysis of the three-dimensional structures of the wild-type (WT) and variant proteins. Our investigation unveiled that the variant protein resulted in significant modifications to the length and overall conformation of the ALS2 protein, impacting both the short and long alsin transcripts (see Fig. 2A and B).

### Splicing analysis of *ALS2* c.1815G > T in the Minigene

Based on the results of splice site prediction software (https://varseak.bio/) (Fig. 2C) and the three-dimensional structure of the mutant protein, we suspect that *ALS2*, c.1815G > T may affect the cleavage of pre-mRNA and thus the function of the gene. Therefore, Minigene assay and RT-PCR analysis were performed to identify the abnormal splicing. Electrophoresis analysis of RT-PCR products showed a about 557 bp band for WT and a shorter about 479 bp bands for MT. Sanger sequencing revealed that this variation causes exon 8 skipping, resulting in a deletion of 78 bp (see Fig. 3C and D).

## Discussion

*ALS2*-related disorder is inherited in an autosomal recessive manner and has been described in individuals from various ethnic backgrounds [12]. Pathogenic variants in *ALS2* are responsible for a retrograde degeneration of the upper motor neurons of the pyramidal tracts. IAHSP is a condition characterized by the gradual involvement of cranial nerves, leading to symptoms such as increased reflexes, persistent clonic lower extremity stiffness, and eventually, upper extremity involvement. As the condition advances, quadriplegia, speech and eating difficulties, dysphagia, and slow eye movements may occur. The disease exhibits significant genetic heterogeneity, and there can be considerable variability within families [13]. In this study, we present the clinical and genetic findings in a chinese family with IAHSP caused by a novel *ALS2* variant. To our knowledge, this is the first report of IAHSP caused by an *ALS2* pathogenic variant (c.1815G > T(p.Lys605Asn)) in China. The variant has not been documented in population and disease databases (PM2-supporting), including 1 K Genomes (https://www.internationalgenome.org/), Human Gene Variant Database (http://www.hgmd.cf.ac.uk/ac/), ClinVar (https://www.ncbi.nlm.nih.gov/clinvar/), and LOVD (http://www.lovd.nl/LTBP-4). The clinical presentation of all affected individuals in this family are consistent with the symptoms of IAHSP (PP4), and the genotype co-segregated with the phenotype in at least one family tested (PP1). A Minigene splicing assay confirmed that the variation causes exon 8 skipping (deletion 78 bp) which met the PM4. The amino-terminal region shares homology with RCC1 (regulator of chromatin condensation factor 1), a known GEF (guanine exchange factor) domain of the Ran family of small GTPases [14]. The c.1815G > T(p.Lys605Asn) variant is located within the RCC1 domain the RCC1 domain. Additionally, bioinformatics tools predict the variant to be deleterious (PP3).

Based on the existing evidence, the variant c.1815G > T(p.Lys605Asn) can be classified as likely pathogenic. This classification is supported by multiple factors, including PVS1_O(a pathogenic evidence code of variable strength),PM2, PP1, PP3, and PP4, which align with the guidelines established by the ACMG [10].

Both of our siblings exhibited normal intelligence initially but experienced motor development regression at the age of 2. Currently, their upper limb muscle tone is normal, while their lower limb muscle tone is increased. They have poor balance and are unable to stand independently. Although they can communicate, their speech is slurred. Additionally, they exhibit abnormal posture, triceps reflex, tendon hyperreflexia, and ankle clonus (+). To further investigate genotype-phenotype correlations, we conducted a comprehensive review of variants reported in various databases (OMIM, Wanfang, CNKI, PubMed), considering both the clinical phenotype and genetic background (refer to Table 1). Our statistical analysis revealed a total of 26 reported variants, with LoF variants being the most common type observed (84.62%). Research suggests that the average age of onset for IAHSP is 1.53 ± 0.53 years, with a male-to-female ratio of 1:0.74. Typically, the disease leads to the loss of walking ability around 0.81 ± 1.57 years of age, with primary clinical symptoms including developmental regression, dysarthria and clonus (100, 95.65, 95.45%). However, the life expectancy of individuals with IAHSP remains unaffected, and cognitive function is preserved. Several studies have demonstrated the genetic heterogeneity of the gene. However, in advanced stages of *ALS2*, there appears to be minimal variation in the observable characteristics [4]. Despite differences in disease severity and variant types, individuals with *ALS2* ultimately develop similar symptoms, including loss of mobility, upper extremity dysfunction, and bulbar symptoms.

**Table 1:** Clinical features and *ALS2* mutations reported in patients with infantile-onset ascending spastic paralysis

Patient	Gender	Age at exam (year)	Age at onset (year)	Loss of ambulation (year)	Upper limb involvement	Dysphagia	Dysarthria	Developmental regression	Clonus	Ocular gaze paralysis	Cognitive impairment	Abnormal Brain MRI	Country of origin	Mutation
**1**	boy	14	1.2	2	+	NA	+	+	+	–	–	+	Kuwait	c.1425_1426delAG(p.E476Gfs*71) [8, 15] (Homozygous)
**2**	boy	6	0.9	NW	+	NA	+	+	+	–	–	+		
**3**	boy	2.5	0.8	NW	+	NA	+	+	–	–	–	NA		
**4**	boy	36	1	NW	+	+	+	+	+	–	–	+	Algeria	c.3619delA(p.M1207*) [16, 17] (Homozygous)
**5**	girl	31	1	NW	+	+	+	+	+	–	–	NA		
**6**	boy	24	1	NW	+	+	+	+	+	–	–	NA		
**7**	girl	18	1.5	4	+	+	+	+	+		–	+	France	c.1472_1481delGTTTCCCCCA(p.V491Gfs*3) [16, 17] (Homozygous)
**8**	girl	23	1.3	5	+	+	+	+	+	+	–	+	Italy	c.2537_2538delAT(p.N846Ifs*13) [16, 17] (Homozygous)
**9**	boy	20	1.5	4	+	+	+	+	+	+	–	+	Italy	c.1007_1008delTA(p.I336Tfs*5) [16, 17] (Homozygous)
**10**	girl	12	1.5	>1.5	+	NA	+	+	NA	+	NA	NA	Pakistan	c.4721delT(p.V1574Afs*44) [18] (Homozygous)
**11**	girl	9	1	NW	+	+	+	+	+	NA	NA	NA	Buchari Jewish- Israel	c.2992C > T(p.R998*) [19] (Homozygous)
**12**	girl	6	1.2	NW	+	+	+	+	+	NA	–	–		
**13**	girl	22	<1	NW	+	–	+	NA	NA	NA	NA	NA	Turkey	c.470G > A(p.C157Y) [20] (Homozygous)
**14**	girl	20	<1	NW	+	+	+	NA	NA	NA	–	+		
**15**	girl	13	0.7	NW	+	+	+	+	+	NA	–	–	Netherlands	c.2143C > T(p.Q715*) [21] (Homozygous)
**16**	boy	8	1.5	NW	+	+	+	+	+	NA	–	–		
**17**	girl	11	0.8	NW	+	+	+	+	+	NA	+	–	Hungary	c.1821_1826insCAGTG(p.E609Afs*9); c.3529G > T(p.G1177*) [22] (compound heterozygous)
**18**	girl	6	<1	NW	–	–	+	+	+	NA	+	–		
**19**	boy	7	1.5	NW	+	NA	+	+	+	–	+	+	Germany	c.1999–2 A > T(p.E724fs*32) [23] (Homozygous)
**20**	boy	17	1	NW	+	+	+	+	+	–	–	+	Italy	c.3836 + 1G > T(p.K1234 fs*3) [24] (Homozygous)
**21**	boy	16	3	NW	+	+	+	+	+	+	–	–	Portugal	c.1425_1428del (p.G477Afs*19); c.145G > A(p.G49R) [25] (compound heterozygous)
**22**	girl	8	<2	4	+	+	+	+	+	NA	–	+	Saudi Arabia	c.2761C > T(p.R921*) [26] (Homozygous)
**23**	boy	4	2	4	NA	+	+	+	+	NA	–	NA		
**24**	girl	19	2	+	+	+	+	+	+	NA	NA	NA	Turkey	c.2351 + 2C > A [27] (Homozygous)
**25**	girl	12	0.5	NW	+	+	+	+	+	NA	NA	NA		
**26**	boy	6	1	NW	+	+	+	+	+	–	–	+		
**27**	girl	4.5	1.5	NW	+	+	+	+	+	–	–	+		
**28**	boy	15	<2	NW	+	NA	+	+	+	NA	+	NA	Pakistan	c.2998delA(p.I1000*) [28] (Homozygous)
**29**	boy	13	<2	NW	+	NA	+	+	+	NA	+	NA		
**30**	boy	16	<2	NW	+	NA	+	+	+	NA	+	–		
**31**	boy	7	<2	NW	+	NA	+	+	+	NA	+	NA		
**32**	girl	18	<2	NW	+	NA	+	+	+	NA	+	NA		
**33**	boy	6	<2	NW	+	NA	+	+	+	NA	+	NA		
**34**	boy	NA	<2	NW	+	+	+	+	+	–	–	NA	Pakistan	c.194 T > C(p.F65S) [28] (Homozygous)
**35**	boy	13	<2	NW	+	+	+	+	+	–	–	NA		
**36**	boy	NA	<2	NW	+	+	+	+	+	–	–	NA		
**37**	boy	22	<2	NW	+	+	+	+	+	–	–	–		
**38**	boy	26	<2	NW	+	+	+	+	+	–	–	NA		
**39**	boy	7	<2	NW	–	–	+	+	+	–	–	–	Pakistan	c.1918C > T(p.R640*) [29] (Homozygous)
**40**	girl	6	<2	NW	–	–	+	+	+	–	–	NA		
**41**	girl	14	2	4	+	NA	+	+	+	NA	–	–	Spanish	c.2944delC (p.Gln982Serfs*19; c.2110C > T (p.Arg704Ter) [30] (compound heterozygous)
**42**	girl	NA	2	NA	NA	NA	NA	+	+	NA	NA	NA		c.1918C > T (p.Arg640Ter); c.145G > A (p.Gly49Arg) [30] (compound heterozygous)
**43**	girl	5	1.5	NW	+	–	+	+	+	–	-	–	Iran	c.1640 + 1G > A [31] (Homozygous)
**44**	boy	13	1	NW	+	+	+	+	–	–	–	–		
**45**	boy	7	1.5	NW	+	+	+	+	+	–	–	–		
**46**	boy	19	1	2	+	+	+	+	+	–	+	+	India	c.3692_3693del(p.Leu1231Glnfs*15) [32] (Homozygous)
**Present study**	girl	11	<2	4	–	+	–	+	+	–	–	–	China	c.1815G > T (p.Lys605Asn) (Homozygous)
	boy	5.6	<2	3.5	–	+	–	+	+	–	–	–		
**rate**	Boy:girl = 1:0.74	13.3 ± 7.62	1.53 ± 0.53	0.81 ± 1.57	89.13%	85.29%	95.65%	100%	95.45%	14.81%	25.00%	46.43%		LoF variants: missense 22:4

*NA* not available or not assessed, *NW* never walked, *LoF* loss-of-function

Currently, there are no successful remedies available for IAHSP. It is recommended to seek support from a multidisciplinary team of specialists, including neurologists, orthopedists, physical therapists, occupational therapists, speech and language therapists, as well as gastroenterologists and nutritionists who specialize in feeding issues.

## Conclusions

In this study, we present the clinical and genetic findings of two Chinese patients diagnosed with IAHSP. The underlying cause of their condition was identified as novel *ALS2* likely pathogenic variants. The identification of these variants, along with the clinical features observed in these patients, contributes to the diversity of genotypic spectrum in IAHSP. Furthermore, it expands the range of phenotypic manifestations observed in individuals of different ethnic backgrounds. These findings hold significant value in terms of genetic diagnosis and future variant-based screening for this disorder.

### Supplementary Information

**Additional file 1.**

**Additional file 2.**

**Additional file 3.**

## Data Availability

The datasets used and/or analyzed in the present study are available from the corresponding author on reasonable request. The sequencing dataset has been deposited in NCBI Sequence Read Archive, and the BioProject ID is PRJNA1018113 (https://www.ncbi.nlm.nih.gov/sra/PRJNA1018113).

## Abbreviations

IAHSP: Infantile-onset ascending hereditary spastic paralysis
WES: Whole exome sequencing
ALS: Amyotrophic lateral sclerosis
JPLS: Juvenile Primary Lateral Sclerosis
GEF: Guanine nucleotide exchange factor
ORF: Open reading frame
UMN: Upper motor neurons
LMN: Lower motor neurons
WT: Wild-type
MRI: Magnetic resonance imaging
