# Supplementary figures and images for: Clinical features and molecular genetic investigation of infantile-onset ascending hereditary spastic paralysis (IAHSP) in two Chinese siblings caused by a novel splice site ALS2 variation
Source: BMC Med Genomics. 2024 Jan 31;17:44. doi: 10.1186/s12920-024-01805-x (PMC10829245; doi:10.1186/s12920-024-01805-x)

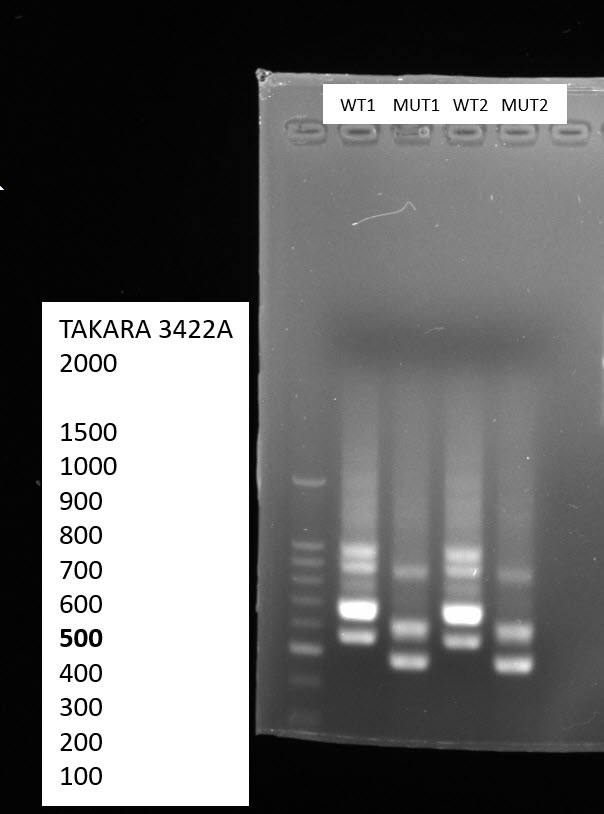

Supplement: Supplementary file 1 — Additional file 1. [file 12920_2024_1805_MOESM1_ESM.jpg]

Sister

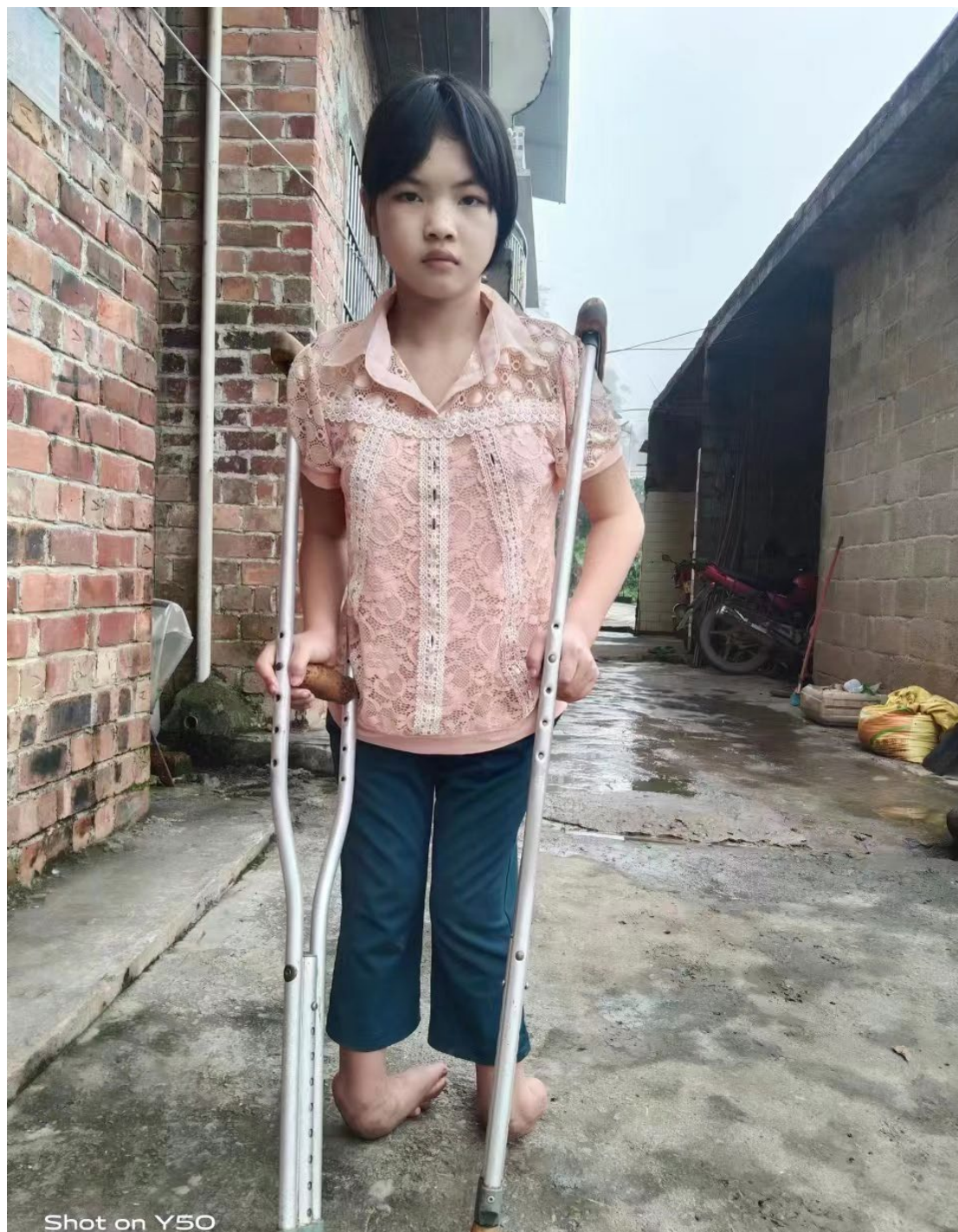

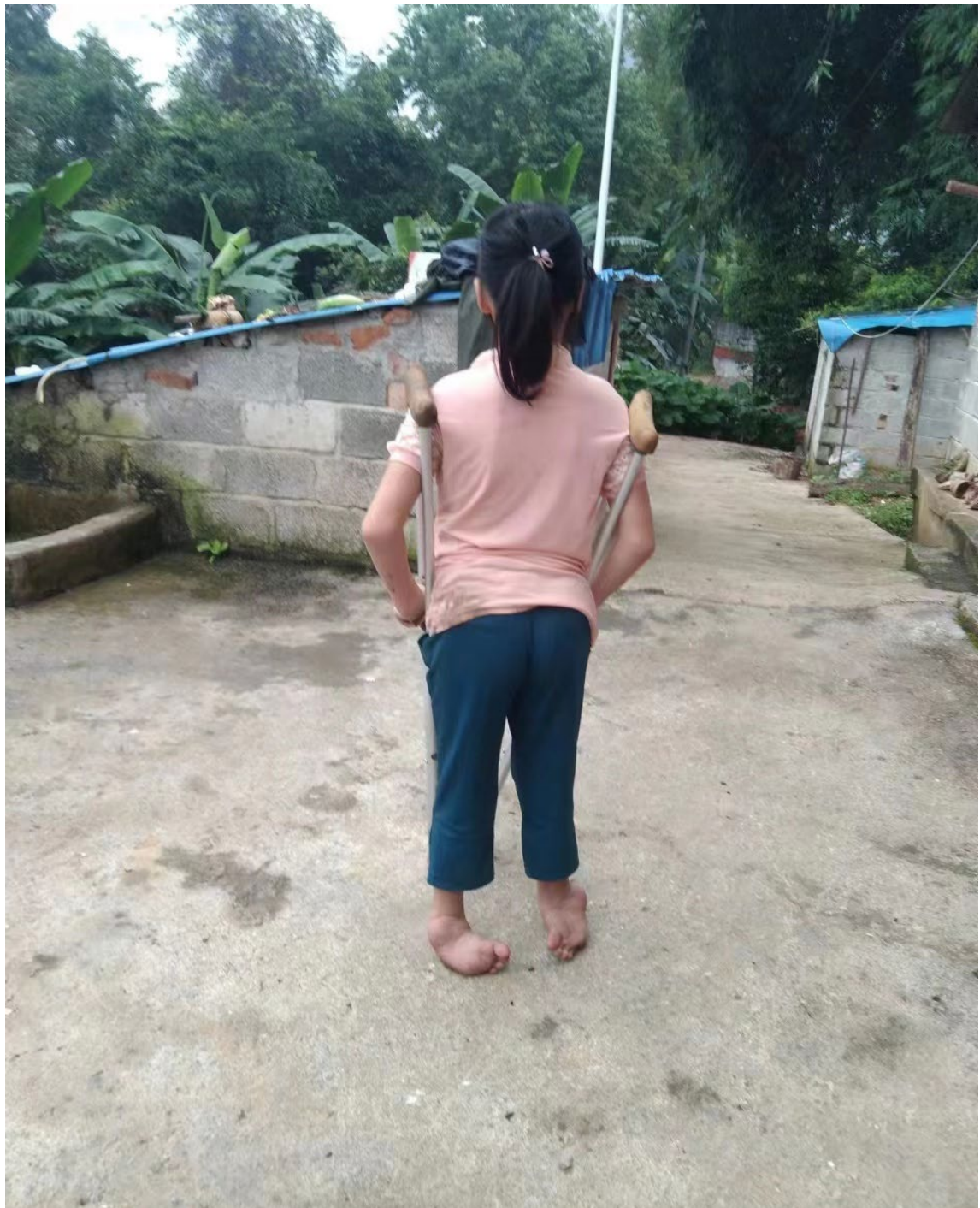

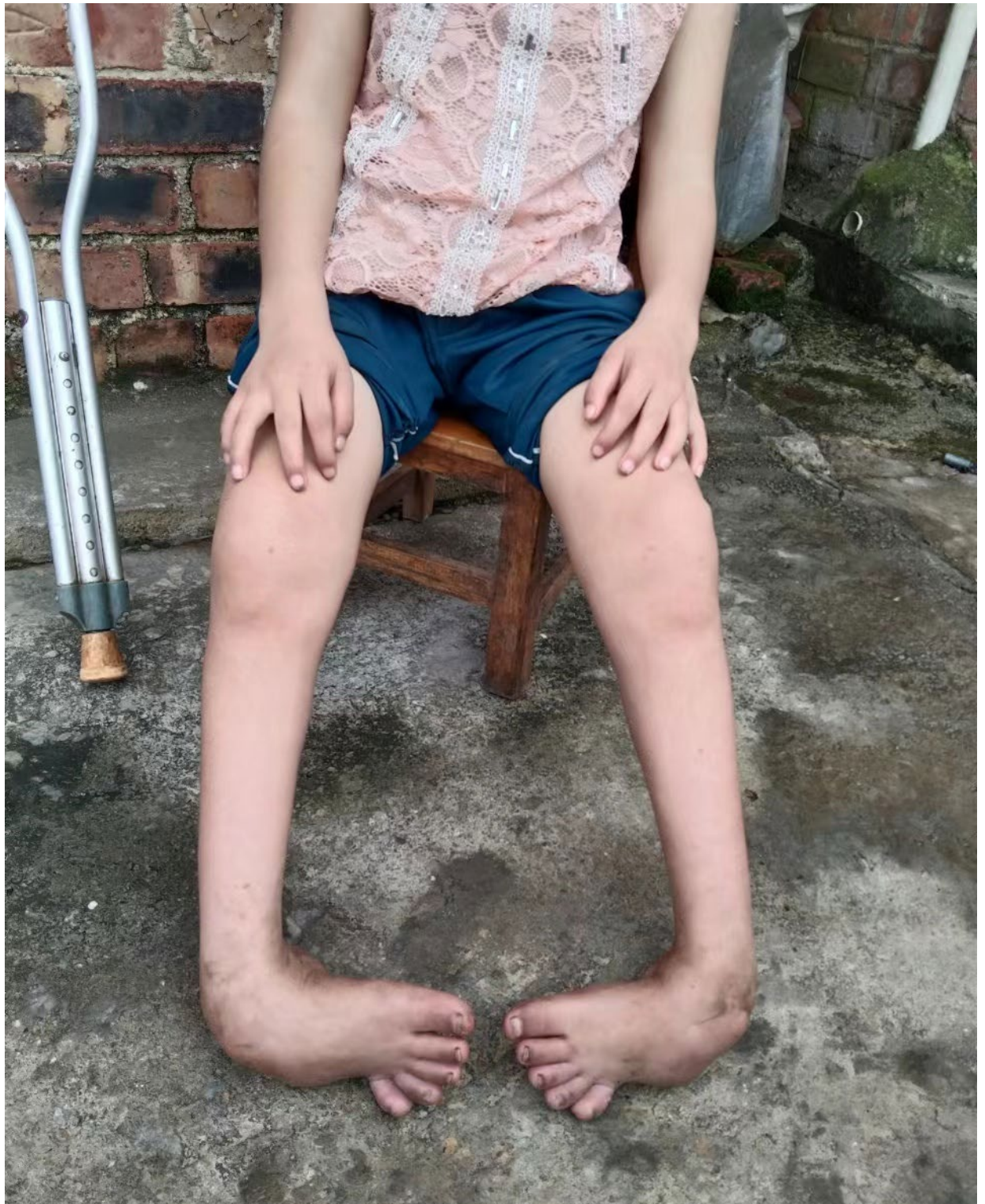

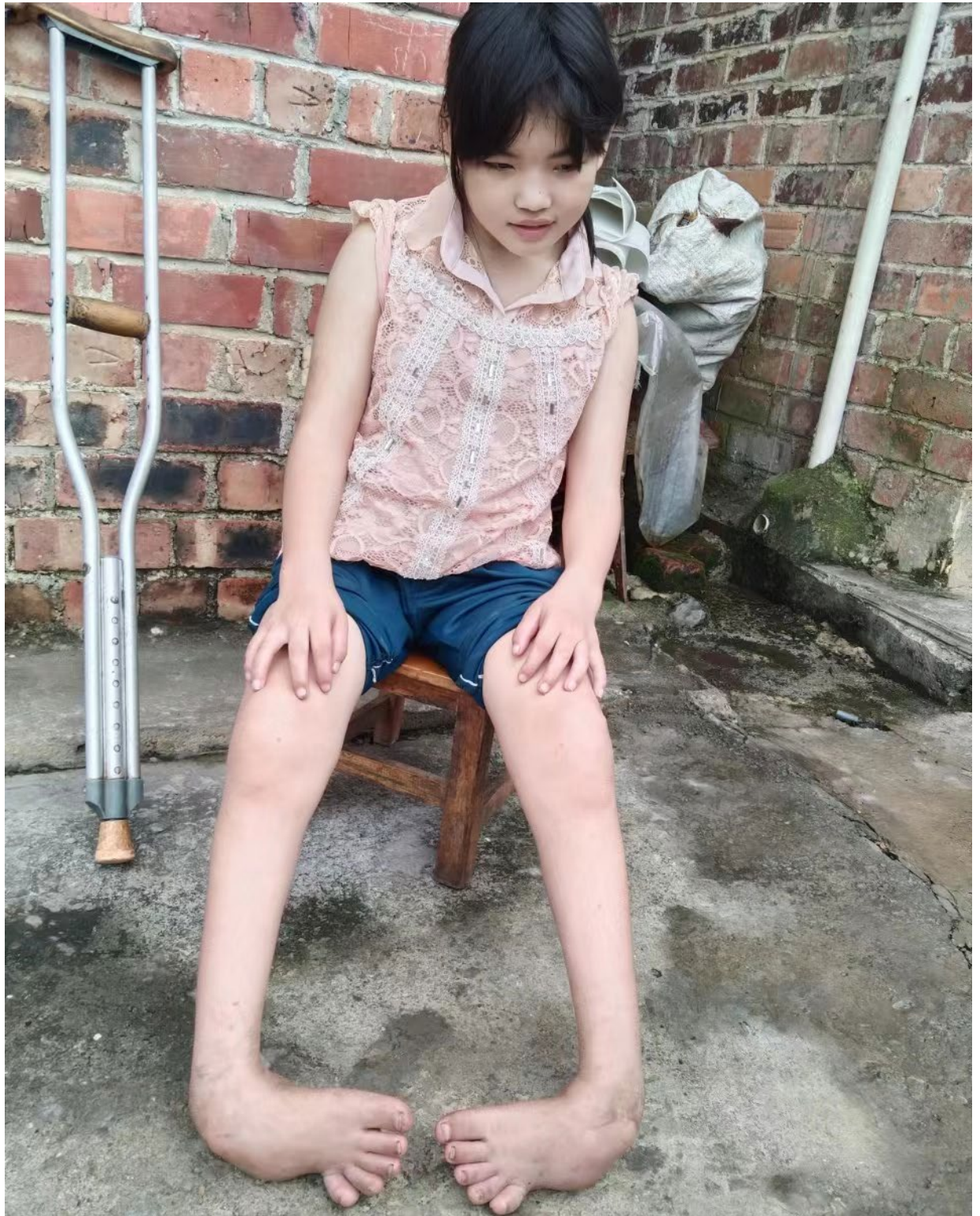

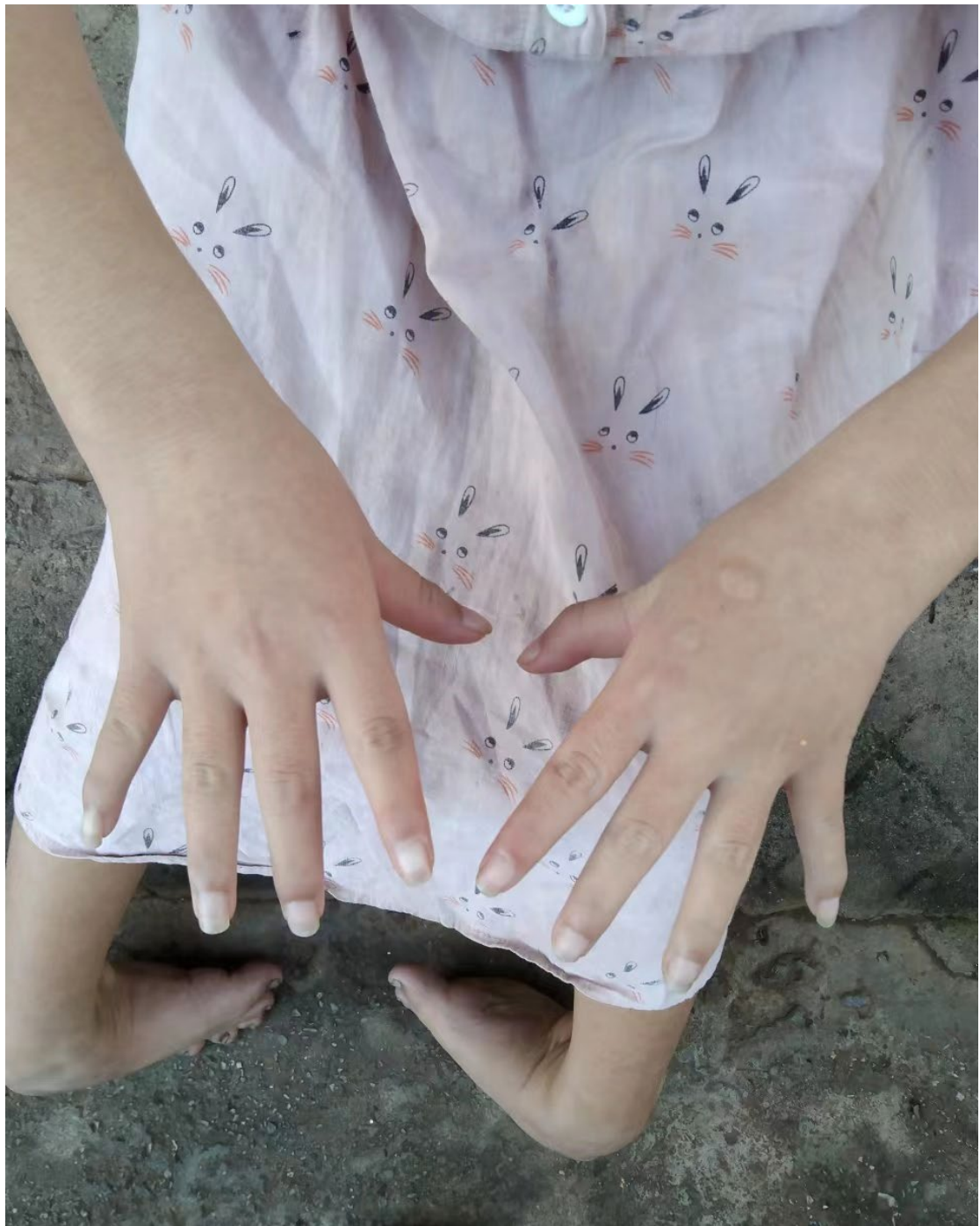

Brother

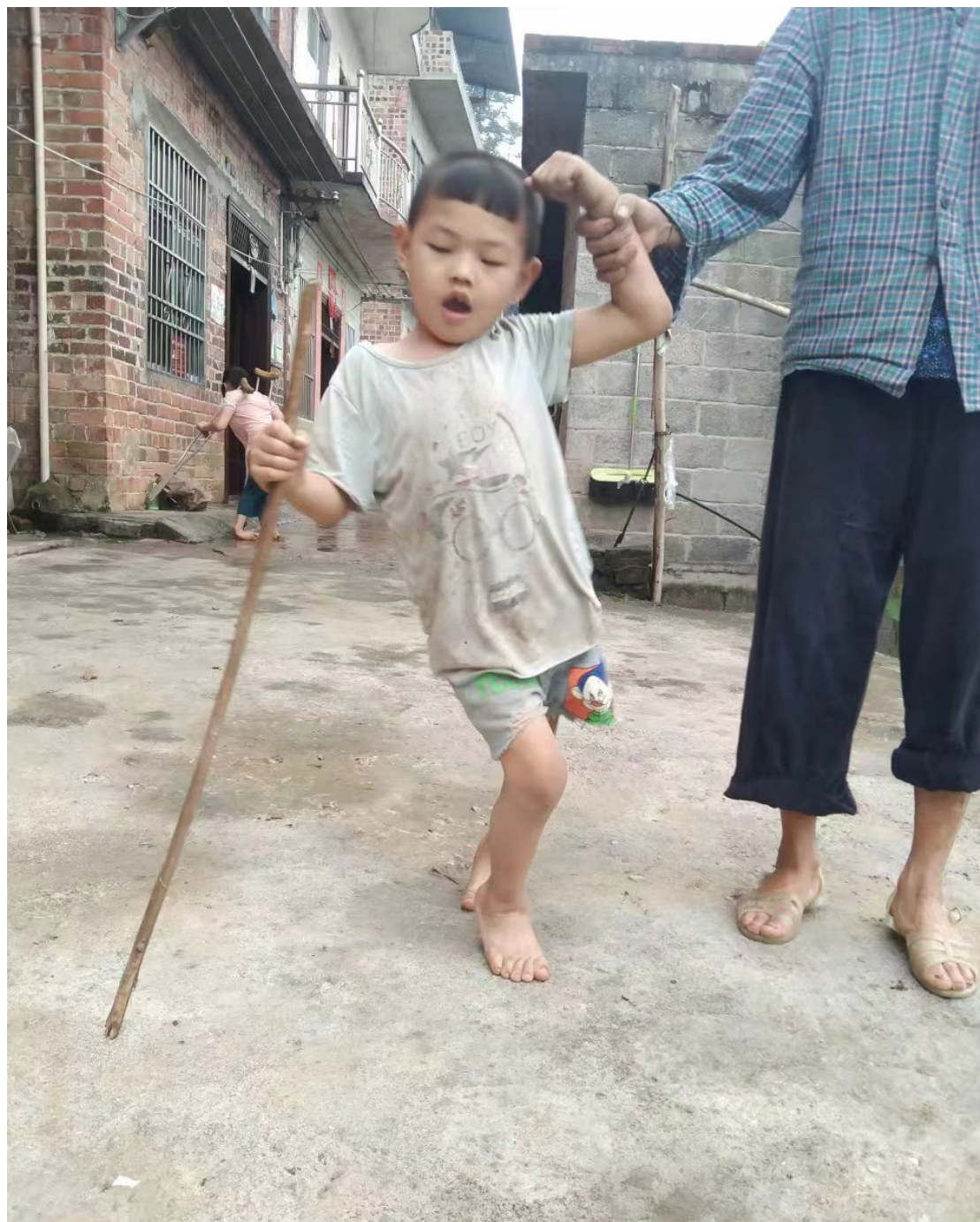

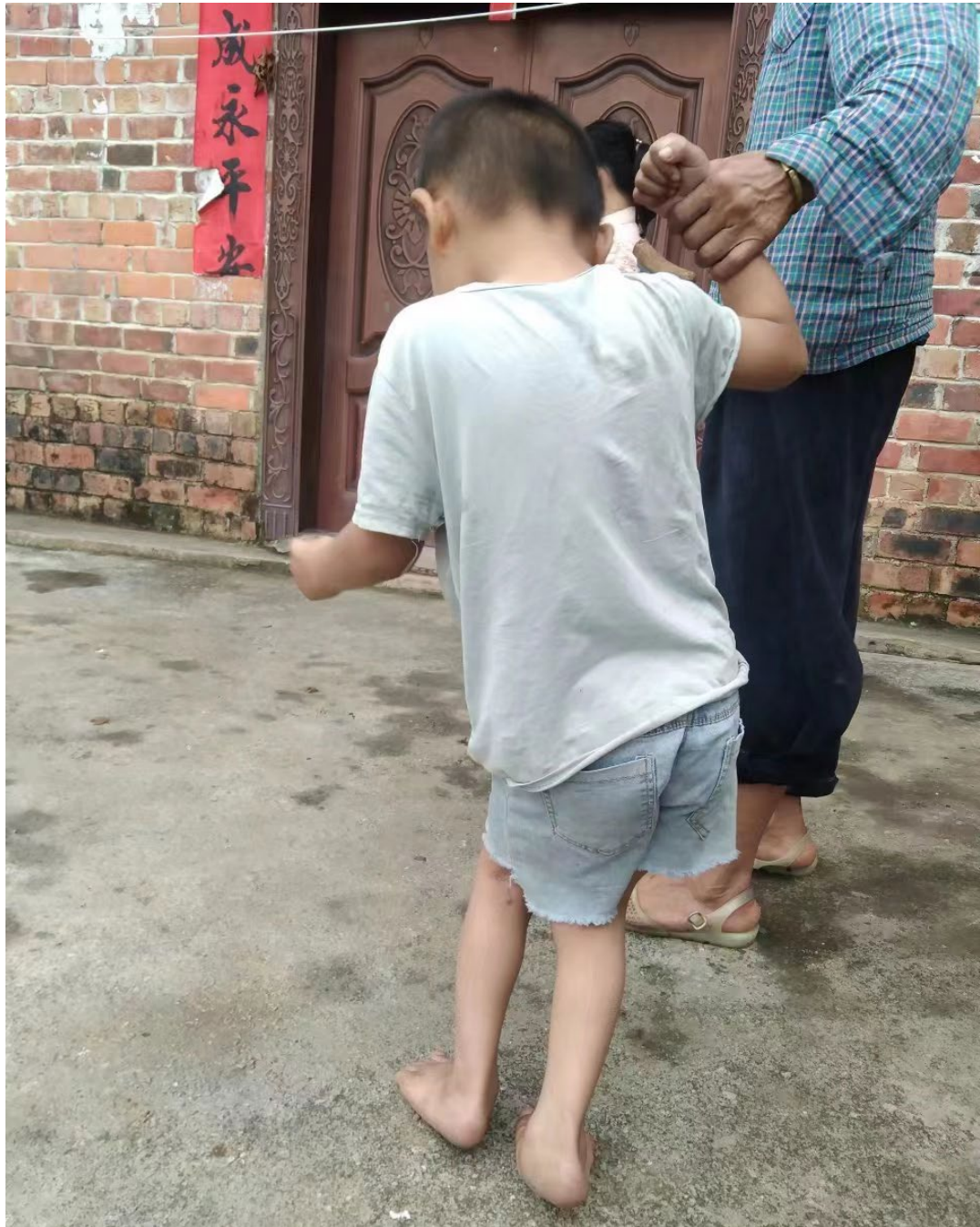

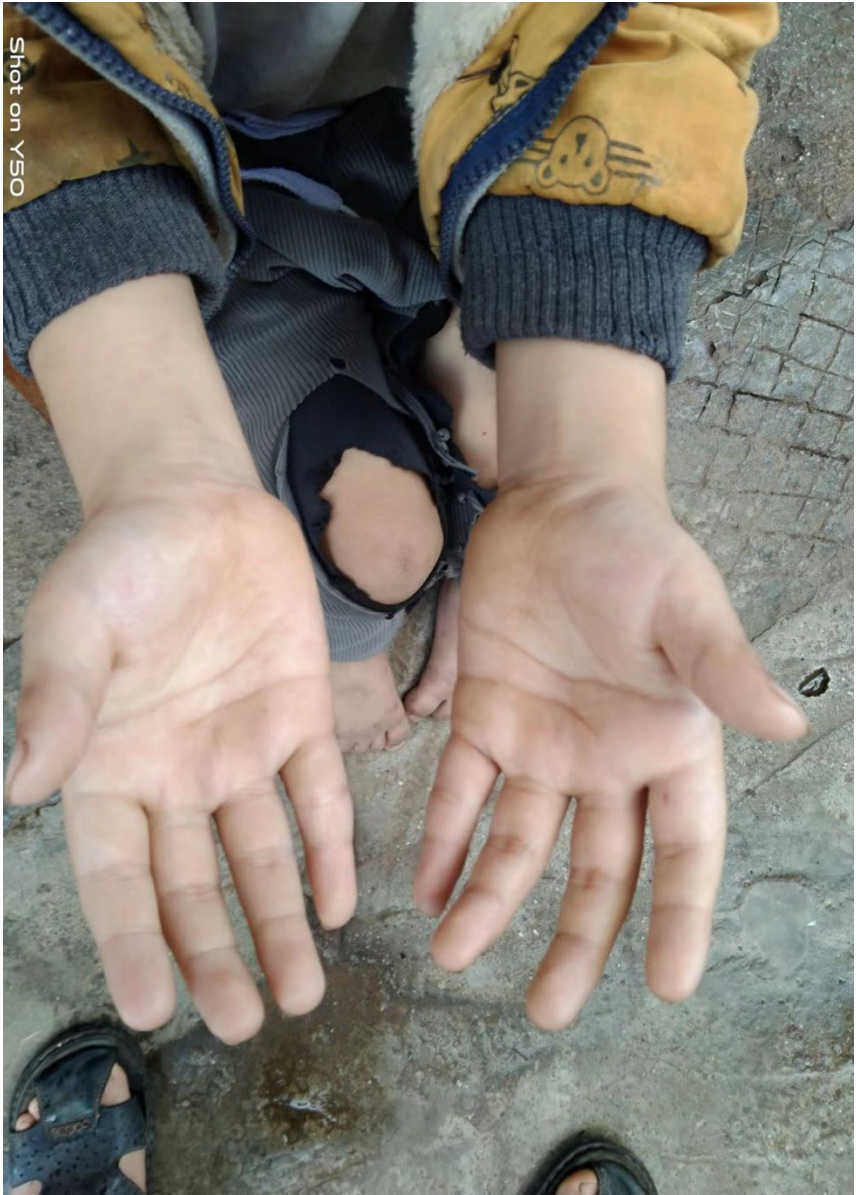

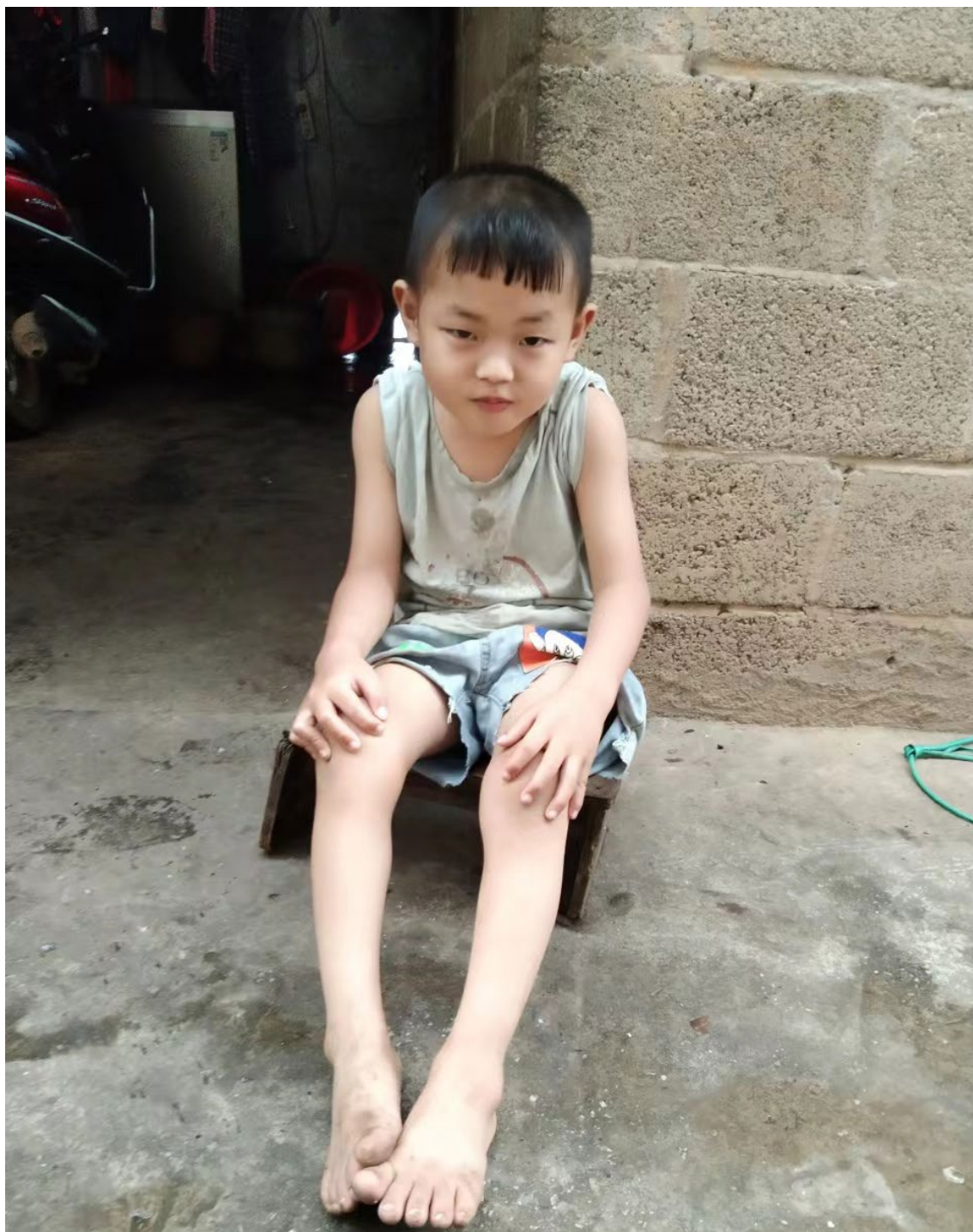

Supplement: Supplementary file 3 — Additional file 3. [file 12920_2024_1805_MOESM3_ESM.pdf]
