# Supplementary material for: Clinical features and molecular genetic investigation of infantile-onset ascending hereditary spastic paralysis (IAHSP) in two Chinese siblings caused by a novel splice site ALS2 variation
Source: BMC Med Genomics. 2024 Jan 31;17:44. doi: 10.1186/s12920-024-01805-x (PMC10829245; doi:10.1186/s12920-024-01805-x)
Supplement: Supplementary file 2 — Additional file 2. [file 12920_2024_1805_MOESM2_ESM.pdf]

ALS2 cDNA seq:

ACTGGGTTGC CAAGCTCGCG CCGGATGCGG AGCGCGGTGC TGCCGGTGGA 50
GCTTCAGGTC TTGATAGACT TTCTGTAAAG AAGGAATGAT TTGGTGATGG 100
AGTGTTCCCA CTGACCGATG GACTCAAAGA AGAGAAGCTC AACAGAGGCA 150
GAAGGATCCA AGGAAAGAGG CCTGGTCCAT ATCTGGCAGG CAGGATCCTT 200
TCCCATAACA CCAGAGAGAT TGCCAGGCTG GGGAGGAAAG ACTGTTTTGC 250
AGGCAGCCCT CGGAGTGAAA CATGGAGTTC TTCTGACTGA AGATGGTGAG 300
GTCTACAGCT TTGGGACTCT TCCCTGGAGA AGTGGACCAG TGGAGATTTG 350
TCCAAGTAGC CCCATTCTAG AAAATGCCCT GGTTGGGCAA TATGTTATTA 400
CTGTGGCAAC AGGAAGCTTC CATAGTGGAG CAGTGACAGA CAATGGTGTC 450
GCGTACATGT GGGGAGAGAA TTCTGCTGGC CAGTGTGCAG TAGCCAACCA 500
GCAGTATGTG CCGGAACCAA ATCCTGTCAG CATTGCTGAT TCTGAGGCCA 550
GCCCTTTGTT AGCAGTCAGG ATTTTACAGT TGGCGTGTGG CGAGGAGCAC 600
ACTCTGGCAT TGTCAATAAG CAGAGAGATT TGGGCATGGG GTACCGGTTG 650
TCAGTTGGGT CTCATTACCA CTGCCTTCCC AGTGACAAAG CCGCAAAAGG 700
TAGAACATCT TGCTGGGCGA GTGGTGCTTC AAGTTGCCTG TGGTGCTTTC 750
CACAGCTTAG CCCTTGTACA ATGCCTCCCT TCCCAGGATC TGAAGCCAGT 800
CCCAGAACGA TGCAACCAGT GCAGCCAGCT CTTGATTACT ATGACTGACA 850
AAGAAGACCA TGTGATTATA TCAGACAGTC ATTGTTGCCC ATTAGGTGTG 900
ACACTGACAG AATCTCAGGC AGAAAACCAT GCCAGCACTG CTCTCAGCCC 950
CTCCACTGAA ACCCTTGACA GGCAGGAAGA AGTATTTGAG AACACTCTTG 1000
TAGCAAATGA TCAGTCTGTT GCTACTGAAC TGAATGCAGT AAGTGCTCAG 1050
ATCACAAGCA GCGATGCCAT GTCCTCTCAA CAAAATGTCA TGGGAACAAC 1100
TGAAATTTCC TCTGCCAGAA ACATACCATC ATACCCTGAC ACCCAAGCAG 1150
TCAATGAATA CCTACGGAAA CTGTCAGATC ATTCAGTAAG AGAGGACTCA 1200
GAGCATGGTG AAAAGCCAGT GCCATCTCAG CCTCTTTTAG AAGAAGCAAT 1250
TCCTAATCTC CACAGCCCGC CTACCACAAG CACCTCAGCC CTAAACAGCC 1300
TGGTGGTCTC TTGTGCATCT GCTGTTGGTG TGAGAGTGGC TGCTACTTAT 1350
GAAGCTGGTG CCTTGTCACT GAAGAAAGTT ATGAACTTTT ATAGTACAAC 1400
CCCTTGTGAA ACTGGAGCTC AGGCAGGCAG TAGTGCCATT GGCCCCGAAG 1450
GTTTGAAAGA TAGCAGGGAA GAACAGGTTA AACAGGAATC AATGCAAGGA 1500
AAGAAAAGTT CAAGTCTTGT GGATATCAGA GAAGAAGAAA CAGAGGGAGG 1550
CAGTCGAAGA CTCTCCCTCC CTGGATTGTT GTCACAAGTT TCCCCCAGGC 1600
TCTTAAGAAA GGCTGCACGG GTGAAAACGA GGACAGTGGT TCTGACCCCC 1650
ACATACAGTG GAGAAGCAGA TGCGCTCCTG CCTTCTCTGA GAACAGAAGT 1700
GTGGACCTGG GGGAAAGGGA AGGAAGGGCA GCTGGGGCAC GGCGATGTTC 1750
TGCCTAGGCT TCAACCGTTG TGTGTAAAAT GTCTGGATGG CAAAGAAGTA 1800
ATCCATCTGG AGGCAGGTGG TTACCATTCT CTTGCACTTA CTGCGAAATC 1850
CCAGGTTTAC TCATGGGGTA GCAATACCTT TGGTCAACTT GGGCATTCCG 1900
ATTTTCCAAC AACAGTTCCT CGTCTTGCAA AGATAAGCAG TGAAAATGGA 1950
GTCTGGAGCA TAGCTGCAGG CAGGGATTAT TCCCTGTTTT TAGTGGATAC 2000
AGAAGACTTC CAGCCTGGGT TATATTACAG TGGCCGACAG GACCCTACAG 2050
AAGGTGACAA CCTTCCAGAG AATCACAGTG GTTCTAAGAC TCCAGTACTT 2100

CTCTCCTGTA GTAAGCTTGG ATATATAAGC AGAGTGACAG CAGGAAAAGA 2150
TAGCTATTTA GCCTTGGTGG ATAAAAACAT TATGGGGTAT ATTGCCAGTC 2200
TCCACGAGTT AGCTACTACA GAAAGACGAT TCTATTCAAA ACTAAGTGAT 2250
ATCAAATCTC AGATTCTCAG GCCTCTTCTC AGTTTAGAAA ATTTGGGCAC 2300
TACAACTACA GTCCAGCTGT TGCAGGAGGT GGCTAGCCGA TTCAGCAAGC 2350
TGTGTTACCT CATTGGTCAG CATGGAGCCT CATTGAGCAG CTTCCTTCAT 2400
GGGGTAAAGG AAGCCAGGAG TTTGGTCATC CTGAAGCATT CAAGTCTCTT 2450
CTTGGATAGT TATACAGAGT ATTGCACATC TATTACAAAT TTCCTGGTTA 2500
TGGGAGGATT CCAGCTTCTT GCTAAGCCTG CCATTGATTT CCTAAATAAA 2550
AACCAAGAGC TGTTGCAAGA TTTGTCAGAA GTGAATGACG AAAACACTCA 2600
GTTGATGGAA ATACTGAATA CTTTGTTTTT CTTGCCAATC AGACGACTTC 2650
ATAATTACGC AAAAGTTTTG CTAAAGCTTG CTACTTGTTT TGAAGTGGCA 2700
TCTCCAGAAT ATCAGAAACT GCAGGATTCC AGTTCTTGTT ATGAGTGTCT 2750
TGCTCTCCAT CTCGGCAGGA AAAGGAAGGA AGCAGAATAC ACACTGGGCT 2800
TCTGGAAGAC CTTCCCCGGA AAAATGACGG ATTCCTTGAG GAAGCCAGAG 2850
CGTCGACTGC TGTGTGAGAG TAGTAACCGA GCCCTGTCTC TGCAGCATGC 2900
TGGGAGGTTT TCCGTGAATT GGTTCATTCT CTTTAATGAT GCCCTGGTCC 2950
ATGCCCAGTT CTCCACGCAC CATGTTTTCC CTCTGGCCAC GCTGTGGGCA 3000
GAGCCACTGT CTGAAGAAGC TGGTGGTGTG AATGGCTTAA AGATAACTAC 3050
ACCTGAGGAG CAGTTCACTC TCATTTCATC TACACCCCAG GAAAAGACAA 3100
AGTGGCTACG AGCTATAAGC CAAGCCGTAG ATCAGGCTTT GAGAGGGATG 3150
TCTGATCTCC CCCCTTATGG AAGTGGTAGC AGTGTTCAGA GACAGGAACC 3200
ACCCATTTCA CGCAGTGCCA AATATACTTT CTACAAGGAT CCTCGCCTAA 3250
AGGATGCCAC CTATGATGGA CGCTGGCTTT CAGGGAAGCC TCATGGCAGA 3300
GGGGTTTTGA AGTGGCCTGA TGGAAAGATG TATTCTGGCA TGTTCAGGAA 3350
TGGCTTGGAA GATGGGTATG GAGAATACAG AATCCCAAAC AAGGCAATGA 3400
ACAAAGAAGA CCATTATGTG GGCCATTGGA AAGAAGGAAA AATGTGCGGT 3450
CAAGGAGTCT ACAGCTATGC TTCTGGTGAA GTATTTGAGG GCTGTTTTCA 3500
AGATAATATG CGTCATGGTC ATGGTCTTCT ACGAAGTGGG AAATTGACGT 3550
CCTCTTCTCC TAGTATGTTC ATTGGCCAGT GGGTAATGGA TAAGAAAGCA 3600
GGATATGGTG TCTTTGATGA TATCACTAGG GGGGAAAAGT ATATGGGAAT 3650
GTGGCAAGAT GATGTGTGTC AAGGGAATGG TGTGGTGGTT ACCCAGTTTG 3700
GATTATACTA CGAGGGCAAC TTTCACCTTA ATAAAATGAT GGGAAATGGG 3750
GTTTTGCTTT CCGAAGATGA TACTATCTAT GAAGGAGAAT TTTCAGATGA 3800
CTGGACTCTT AGTGGAAAGG GAACACTGAC TATGCCAAAT GGAGACTACA 3850
TTGAAGGTTA TTTTAGTGGA GAATGGGGAT CTGGGATAAA AATCACTGGA 3900
ACCTACTTCA AACCTAGTCT ATATGAGAGT GATAAAGACA GACCTAAAGT 3950
TTTCAGGAAG CTAGGAAACC TGGCAGTGCC AGCTGATGAG AAGTGGAAAG 4000
CGGTGTTTGA CGAATGTTGG CGCCAACTGG GCTGTGAGGG CCCAGGCCAA 4050
GGGGAAGTTT GGAAAGCATG GGACAATATT GCTGTGGCCT TGACCACCAG 4100
TCGGCGCCAG CACAGAGACA GTCCAGAAAT ACTGAGTCGT TCACAGACTC 4150
AGACACTAGA GAGTTTGGAA TTCATTCCAC AGCATGTTGG TGCCTTCTCT 4200
GTGGAGAAAT ATGATGACAT CAGGAAATAT TTAATAAAGG CCTGTGACAC 4250
TCCTCTGCAC CCCCTGGGCA GGCTTGTGGA GACACTGGTT GCAGTGTATA 4300

GAATGACATA CGTGGGCGTA GGAGCCAACC GCAGGTTATT GCAGGAGGCT 4350
GTAAAGGAGA TTAAGTCCTA TCTTAAGCGA ATTTTCCAGC TGGTGAGGTT 4400
CTTATTTCCT GAGCTGCCTG AAGAAGGCAG CACAATTCCT CTCTCTGCTC 4450
CTCTGCCAAC CGAAAGGAAG TCTTTTTGCA CTGGGAAGTC AGATTCCCGA 4500
TCTGAATCAC CAGAGCCAGG TTATGTAGTA ACGAGTTCTG GATTATTGCT 4550
TCCTGTGCTG CTACCTCGGC TCTACCCACC GCTGTTTATG CTTTATGCTT 4600
TGGATAATGA TCGCGAGGAA GACATTTACT GGGAATGTGT CCTTCGACTA 4650
AATAAGCAGC CAGATATTGC TCTCCTGGGC TTTCTTGGGG TGCAGAGGAA 4700
ATTTTGGCCA GCAACCTTGT CAATCCTTGG AGAGAGTAAA AAGGTTTTGC 4750
CAACCACGAA AGATGCTTGT TTTGCCTCAG CAGTAGAATG TCTGCAGCAG 4800
ATCAGCACAA CATTTACCCC ATCAGACAAA CTTAAGGTCA TCCAGCAGAC 4850
TTTTGAGGAG ATCTCTCAGA GTGTCCTGGC GTCACTCCAC GAAGACTTCT 4900
TGTGGTCCAT GGATGACTTG TTTCCTGTTT TCTTATATGT GGTGCTACGG 4950
GCCAGGATTA GGAATTTAGG CTCTGAGGTA CACCTCATTG AGGATCTAAT 5000
GGACCCCTAT CTTCAGCATG GGGAACAGGG TATAATGTTC ACCACCTTGA 5050
AGGCATGTTA CTACCAGATT CAGCGTGAGA AGCTTAACTA GGCTGCATAA 5100
CAGCTTGAAA ACTGGATTAT CTACTACAGA GTGTTATAAC ACCATCTGGA 5150
GTCTTCCTGT AGTGGCAAAA AAGAACAGTG TTGAAATTGG AAAGGACTTT 5200
GTGTTATTTA GGTTGTTAGA ATGAGCCTTA CCAATAATAA GAGCCCTGAG 5250
CCCAGAAAAA AGGACTGTAT AGTTTAAAGG GAGGATTGAA AGGGAGGTAA 5300
AAAATCAGAT TAGACCAGTT CTTGGCCTAT GATAAGTTCC AAAAATACCA 5350
TTTATCTACT ATTTGAAAAA AGAAGAGGAT ATCCCTTCCT ACAGTAAAGG 5400
GTATGTCAGC TACATGAAGT TGTAAGAAAA GCTTCCAGTA GAGCTTCTTA 5450
TATTAAAGAA GTTGATGGAT ATTTTTGAAT TTCTGGTTTG CCTGAATCCA 5500
CCTGCAGTTA CCCCGATCCG TTTGCAAGAA CCAGATCGTA CTTGAAACTA 5550
TAGTGGCCAC ACTCTGCCTT CCTGAGTCCC TTCCAGTCAT GTGTGCATCA 5600
TGTCTCTTTG CCAAGGGAGG GGAGAAAGGA ACTTTTAAAC TGCAGTTTTA 5650
ACTTTTTCTA AGCTGTTTCT TGATGGGAGA GGTTCTGTGC AAAACTACCA 5700
CATTCTGTCC CCAAAATGTG GAATGCATCC AAATAGGAGT CTTCTGCCTC 5750
TTAACTTAAA AGAACATAGG AATTTTGTTT TTGGTTTCTT TATCATGCTA 5800
CAGAGAGTGA ATACACTGGA ATTCAGACAC CGACTCTGAG CTGCTAGGAA 5850
CCTCATTTGT CCATGTGCAA ACGCTGTATT CCAAGGCCTG TGAATGGCAG 5900
CCTGAGGAAG TTTTGCATGC AGGCTGTGTT TTCGAGCAGG ACTAACAACT 5950
GGGAAATAAG CAAAAAACTG CATCGATCCC CAGCCTGGTG TTGTTCTTCC 6000
CTATACTTCA CACTGAACTC AGGATGGGAA GAAAAAGGAA ACAAGCTTTG 6050
GCTTTTTCCA TCTCAAAAGT ATTGTGGCAC CTCAACATTT CAGTGTTTTG 6100
CTTTTTAAAA AATGCCCTAT TGTAAGTTGT TGGTTTATAC TGTATAAGTA 6150
ACACTAGTAG CTGTTTTGAA TAACATAGGT GCTCTTCCTC ATCTCATCTC 6200
CTACACCGTG GTGAGCATAC AGAGTGTCCT GATTTGTGTT AAGTGACTGA 6250
GAAGATGTTA ATTACTTTTG AAAAAGGATC ATGGTTTTTG CTCTACTTTA 6300
TAATCAAGAC AAGTGTTTAT TAAAATACTG TTTTGGAATG TTGGCTGTAA 6350
TGTAACAGCA ATTTTCATAA TAAAAGGCAT TCATCTTTAT GTGGTTGTTT 6400
TATGATTGTA GAAGAAATGA ACAGGGCAGC CTAAAAGATG AAAGGAAACC 6450
CAGACTTAGT ATTTCTTTAG CCCACATTGT AATCCATGCC TCTAAGTATT 6500

GGCCTCAAAA GTCCCTCTGG GGTTTTAGAA ATCTCTGTCT TTAAAATAAA 6550
CGTAAACCAA TAGTTATAGC ACCCTTTCTA GCAAAGAGCA AATTAACTTT 6600
CTGACCCCAA TAACCCAAGG ACACGGGTCT CCAAAGAAAT CTATTATTTC 6650
TTTGAATAAA AAGCATATTT TGATA

Mutation seq:

GGACTCACTATAGGGAGACCCAAGCTGGCTAGCGTTTAAACTTAAGCTTGCTTCAACCGTTGTG
TGTAAAATGTCTGGATGGCAAAGAAGTAATCCATCTGGAGGCAGGTGGTTACCATTCTCTTGCA
CTTACTGCGAAATCCCAGATAAGCAGTGAAAATGGAGTCTGGAGCATAGCTGCAGGCAGGGAT
TATTCCCTGTTTTTAGTGGATACAGAAGACTTCCAGCCTGGGTTATATTACAGTGGCCGACAGGA
CCCTACAGAAGGTGACAACCTTCCAGAGAATCACAGTGGTTCTAAGACTCCAGTACTTCTCTCC
TGTAGTAAGGGATCCACTAGTCCAGTGTGGTGGAATTCTGCAGATATCCAGCACAGTGGCGGCC
GCTCGAGTCTAGAGGGCCCGTTTAAACCCGCTGATCAGCCTCGACTGGGCCTTCTAAGGTGGAA
TTCTGCAGATATCCAGCACAGTGGCGGCCGCTCGAGTCTAGAGGGCCCGTTTAAACCCGCTGAT
CAGCCTCGACTGTGCCTTCTAA

Wild type seq:

TGATTATACGACTCACTATAGGGAGACCCAAGCTGGCTAGCGTTTAAACTTAAGCTTGCTTCAAC
CGTTGTGTGTAAAATGTCTGGATGGCAAAGAAGTAATCCATCTGGAGGCAGGTGGTTACCATTC
TCTTGCACTTACTGCGAAATCCCAGGTTTACTCATGGGGTAGCAATACCTTTGGTCAACTTGGGC
ATTCCGATTTTCCAACAACAGTTCCTCGTCTTGCAAAGATAATCATTGAAAATGGAGTCTGGAGC
ATAGCTGCAGGCAGGGATTATTCCCTGTTTTTAGTGGATACTGAATACTTCCGGCCTGGGTTATA
TTACAGTGGCCGACAGGACCCTACGAAAGGTGACAACCTTCCGGATAATCACAGTGGTTCTAA
GACTCCAGAACTTCTCTCCTGTGGTAAGGGATCCACTGGTCCAGTGTGGTGGAATTCTGCAGAT
ATCCAGCACAGAGGCGGCCGCTCGAGTCTAAAGGGCCCGTTTAAACCCGCTGATCAGCCTCCA
CTGGGCCTTCTAAGGTGGAATTCTGCAGATATCCAGCACAGTGGCGGCCGCTCGAGTCTAGAG
GGCCCGTTTAAACCCGCTGATCAGCCTCGACTGGGCCTTCTAA

The yellow color is the missing base in the patient's *ALS2* gene.
